# A Neighborhood Grid Clustering Algorithm for Solving Localization Problem in WSN Using Genetic Algorithm

**DOI:** 10.1155/2022/8552142

**Published:** 2022-06-28

**Authors:** Junfeng Chen, Samson H. Sackey, James Adu Ansere, Xuewu Zhang, Altangerel Ayush

**Affiliations:** ^1^College of Internet of Things Engineering, Hohai University, Changzhou 213022, China; ^2^School of ICT, Mongolian University of Science and Technology, Ulaanbaatar 13341, Mongolia

## Abstract

Finding the location of sensors in wireless sensor networks (WSNs) is a major test, particularly in a wide region. A salient clustering approach is laid out to achieve better performance in such a network using an evolutional algorithm. This paper developed a clustered network called neighborhood grid cluster which has a node assuming the part of a cluster center focused in every grid. Grid-based clustering is less difficult and possesses a lot of benefits compared to other clustering techniques. Besides, we proposed a localization algorithm that centers around assessing the target area by considering the least estimated distance embedded with the genetic algorithm. Performance standards incorporate the energy representation, connectivity stratagem, and distance measure as fitness functions that assess our localization problem to demonstrate its viability. Simulation results confirm that our approach further improves localization accuracy, energy utilization, node lifetime, and localization coverage.

## 1. Introduction

A wireless sensor network (WSN) comprises hundreds or thousands of energized nodes that are dissipated in the surroundings. Nodes persistently sense the information and engender it through the surroundings [1]. They work cooperatively to process and course sensor information. These sensor nodes send information streams to ground stations either occasionally or in light of occasions, and the ground station sends the information to the objective node. One of the issues in WSN is the means by which to make an efficient organizational design among these nodes [2–4]. Localization plays a significant function. WSN localization is the method involved with assessing the areas of sensors concerning some nearby or worldwide coordinate framework by utilizing the information between sensor nodes estimations like distance or potential point [5]. Some localization methods such as hop count and approximate point-in-triangulation test (APIT) do not even need these estimations; however, these methods are extremely inclined to mistakes because of hindrances in the arrangement area and are consequently least precise. Since the crucial benefit of WSNs is the capacity to deploy them in an impromptu way, it is not plausible to arrange these nodes into groups of predeployment.

Because a sensor node's energy supply is so low, it is unable to send estimates directly to the ground station. The network is divided into small groups known as grids (clusters) to decrease the amount of energy used for communication [6, 7]. Every grid has a grid leader, which is the center of the grid (cluster center). Cluster head determination/assignment is a critical task in WSN clustering [8] and is a task for ideal nodes. The key objective is to sort out nodes into groups to diminish the energy utilized for communication between the individuals and the ground station. Network-based grid clusters isolate the multilayered information space into a given number of cells, and then, at that point, clustering activity is placed on it [9]. These techniques cycle quickly, and in light of the fact that the speed is insignificant to the quantity of information objects, it depends on the quantity of cells isolated in the information space.

Clustering is an unaided artificial intelligence (AI) strategy, which is great at managing massive, unlabeled, and complicated information [10]. The aftereffects of group analysis state that components of groups are portrayed by the most extreme similarity, and there may be greater similarity among every WSN cluster. Subsequently, clustering methods are the productive ways of managing the previously mentioned issues and are broadly acknowledged in the field of data mining [11]. The neighborhood grid cluster (NGC) can understand data clustering naturally and adapt by means of self-assertive shape information deftly. Along these lines, to optimize energy, clustering is utilized in a remote sensor network to build the system lifetime of the sensor node [12]. Clustering is thought of as a high-dimensional type of network shaping grid clustered in WSN. The essential thought of the framework for grid structures is to partition the detected region into equivalent estimated virtual squares where every grid is considered as a group with one cluster center (CC) in each cluster.

In this research article, algorithms developed for localization are utilized to limit the mistake of arbitrarily dispersed WSNs through some modest number of anchor sensors, and in any case, the ideal deployment areas for these sensors will be found by understanding the evolutionary algorithm. The quantity of associated beacon nodes is one of the main elements influencing the system accuracy for the localization algorithm, whether or not it is the range-based estimation or range-free factors [13, 14]. Enough-associated known nodes permit more prominent position exactness. Be that as it may, the techniques to further develop the location predictor by expanding the quantity of associated known sensors in the network are inaccessible. Along these lines, we have expected deployment of nodes: location mindful known nodes and uninformed location nodes which can be accessed with the assistance of known nodes.

In summary, the principle impact of this paper is summed up as follows:

1. A salient clustering method based on a neighborhood grid cluster is proposed to solve localization problems in sensor networks.
2. The algorithms' efficiency and reliability are optimized by implementing the optimization process via the genetic algorithm (GA) approach considering energy consumption, connectivity between nodes, and the Euclidean distance model as our fitness function.
3. This includes thorough comparisons between our optimized proposed NGCGAL solution and the already existing CGAL, weighted CENTA, CENTA, and DV-Hop-based algorithms. In our comparison, the results prove the better usefulness of the proposed NGCGAL by minimizing the localization error and energy consumed and maximizing the number of alive network nodes and network coverage.

The remainder of the paper is coordinated as follows: Section 2 exhibits the related works on localization methods with some clustering approaches and also the use of the genetic algorithm.  Section 3 presents the system implementation and analysis of the detailed steps including our clustering approach, genetic algorithm, and fitness approach.  Section 4 displays simulated graphs of the proposed approach with other baseline algorithms. In Section 5, conclusions are conveyed.

## 2. Related Works and Background

In this segment, we mainly present the related concepts of clustering and the construction of localization principles and analyze the with the use of the genetic algorithm.

### 2.1. Related Localization Methods

There has been an enormous collection of research on localization for WSNs throughout the latest few years [15]. El. Alami and Abdellah [16] presented a design architecture in WSN and IoT to minimize energy consumption [16]. Several tasks performed by objects in the network are affected by energy depletion. Therefore, minimization of energy consumption is proposed for an efficient routing process. The authors utilized GA to model a system that enables data and knowledge transfer, share, and reuse of intelligence systems [17]. Lee and Teng [18] proposed a hierarchical clustering design for energy conservation in WSN. However, mobile networks suffer packet loss due to the movement of nodes in the network design [18]. Wang et al. [19] proposed a method to handle coverage rate within multiple mobile sinks for trajectory scheduling in large-scale wireless sensor networks [19]. Improved particle swarm optimization (PSO) and GA were implemented to solve optimal coverage rate and scheduled moving trajectory for multimobile sinks, respectively. Node clustering is a powerful strategy for extending the network lifetime. The researchers proposed a clustering design to manage the load imposed on clusters around the sink [20]. The network clustering technique in view of the geological area of the sensors has concentrated cluster heads (CHs). All the noncluster head nodes are at the least distance to the CH. For intercluster communication, the CH information data contain the control packet for route buildup. Kumaravel and Panneerselvam solved WSN network lifetime and data transmission delay problems [21]. The WSN design used multiobjective optimization (MOO) algorithm and effective CH picking through organized cluster formation. Consequently, this procedure decreases the use of energy while communicating and working on the lifetime of the network. In the grid-based clustering algorithm, sensor nodes coordinate so that inactive nodes go into the resting mode, and this turns to preserve energy. Intra- and intertransmission is impacted by cluster size. Grid clustering schemes are well known because of their straightforwardness and uniform dissemination of nodes.

He et al. [22] researched the introduction of kernel regression to node localization of anisotropic WSN [22]. Simulation results solved location accuracy and stability using radial basis kernel-based G-LSVR and polynomial-kernel-based P-LSVR. Clustering techniques in view of grids are STING (statistical information grid), CLIQUE (clustering in the quest), and WaveCluster which significantly lessen the simulation runs, yet the results of clustering are sensitive to the quantity of grids in the networks. The authors proposed a localization scheme based on reliable anchor pair selection (RAPS) and quantum-behaved salp swarm algorithm (QSSA) in the anisotropic network [23]. The methodology proposed by the authors further extended the network lifetime and diminished energy utilization by breaking down the cluster size that is done on the most extreme command. To adjust energy utilization, staying away from direct transmission through the indistinguishable dispersion of CHs and energy of node is imperative. Kaur et al. [24] proposed an algorithm based on DV-hop with weighted centroid in WSN localization [24]. DV-hop and weighted centroid DV-hop reduce cost with no additional hardware requirement.

Nithya and Jeyachidra [25] used an artificial bee colony (ABC) algorithm for deploying and selecting anchor nodes for a better coverage area [25]. They further proposed a fitness function to handle the localization error using the bat optimization algorithm (BOA). The authors discussed the trajectory of the beacon node with the influence of accuracy, time, and efficiency for the localization algorithm [26]. The cosine-rule-based localization (CRL) algorithm uses received beacon nodes' position and distance obtained from RSSI to intersect one point by enabling unknown sensors to locate themselves with better accuracy. Han et al. [27] proposed a method using a single anchor node in localization to minimize localization delay [27]. The group of mobile anchor nodes (GMAN) for path planning design a trajectory to help the entire network using three mobile anchor nodes. Xu et al. [28] proposed (guaranteeing surveillance quality with the minimal number of active sensors) a GSMS approach which initially divides the entire area into a few equivalent estimated grids and afterward computes the sensing probability by detecting the likelihood of every active sensor in the observed region [28]. To work on the nature of the bottleneck grids, GSMS earlier assigns the node likely to be involved the highest in the bottleneck network, targeted at limiting the quantity of dynamic sensors that partake in the cover set protection.

### 2.2. Genetic Algorithm

Optimization refers to finding the values of inputs in such a way that we get the “best” yield values. The meaning of “best” changes from one problem to another; however, in numerical terms, it refers to amplifying or limiting at least one objective function, by fluctuating the input factors [29]. A genetic algorithm (GA) is a metaheuristic algorithm that is propelled by the bionetwork. GA is utilized for optimizing problems where we need to limit or augment a given fitness function under a bunch of requirements. GA is typically utilized when the search domain is excessively enormous, and an answer could not be carved out in a sensible time. The GA begins with an arbitrary populace as first generation, and the individual in each stage is assessed by their fitness. Fitness is determined by the main function being utilized for tackling the problem. The fittest individual is chosen stochastically from the current populace, and afterward adjusted by crossover, and mutated to shape the next generation, where new individuals are assessed by their fitness value. The operators are utilized by the GA to help in producing an excellent solution. The mutation is the best operator that GA utilized for getting away from nearby minima [30]. Furthermore, a fitness function is utilized to assess the fitness of the individuals after every iteration. Genetic algorithms have been used to solve optimization problems in the fields of game hypothesis, data mining, mechanical technology, MANETs, engineering planning, etc.

## 3. Proposed Approach: Genetic Algorithm and Clustering Approach for Localization in WSN

### 3.1. System Assumptions

The WSN is expected to consist of *N* sensor nodes deployed haphazardly in the sensing area in a two-layered dimension with no focal control. The sensor networks are addressed by *G*(*V*, *E*) in which *V* defines the arrangement of vertices and *E* portrays the arrangement of edges. The location of nodes is predefined ahead of time utilizing a GPS switchboard operator known as beacon nodes kn. The available sensor nodes are perceived by unknown nodes unk whose locations are assessed utilizing beacon sensor. The unknown target nodes whose location is found in the first simulation run will go about as a collaborator node and give help to all anchor nodes for finding the position of left over unknown nodes. The system framework listed below is expected to be in our research article. The size of the network model is defined as |*N*|=|kn+unk|.

1. The nodes in grid network are fixed
2. The WSN is partitioned into a framework of grids of equivalent sides
3. They are energy-reserved, and the batteries are not battery-powered
4. Nodes are not dealt with after deployment
5. A principal node is appointed to every grid, called grid cluster center
6. The ground station is situated externally in the surveillance area and is not energy required
7. At first, all nodes have a similar energy level; however, the energy utilization changes in light of transmission distance
8. Both cluster center and individual nodes utilize single-hop communication
9. Anchor nodes have distinctive ID, and their positions are known

Each well-defined sensor node intermittently sends their raw estimations to their cluster centers; every CC performs proper application explicit data aggregation activity to create a more elevated level message.

The Euclidean distance *d*_*kn*_ in a WSN is the calculation of where a specific node's location is. Let (*p*, *q*) is the position of an unknown target node and (*p*_kn_, *q*_kn_) be the place of the kn^th^ anchor node. The distance between these nodes is expressed by the equation as

(1) $$\begin{array}{c} d_{\text{kn}}=\sqrt{{\left(p-p_{\text{kn}}\right)}^{2}+{\left(q-q_{\text{kn}}\right)}^{2}}. \end{array}$$

The objective function *f*(kn, unk) of our localization is mathematically formulated in (2): *n* denotes the total number of known nodes, *m* denotes the number of unknown nodes, *d*_kn_ explains the measured distance between a known and an unknown node, *w*_*i*_ represents the weighted value calculated (when known node and an unknown node are not neighbor *w*_*i*_  = 0), and *d*_unk_ is the calculated distance between a node and another node's position.

(2) $$\begin{array}{c} f\left(\text{kn},\text{unk}\right)=\min\sum_{\text{unk}=1}^{m}\sum_{\text{kn}=1}^{n}w_{i}{\left(\left|d_{\text{kn}}-d_{\text{unk}}\right|\right)}^{2}. \end{array}$$

Localization error LE_*i*_ is described as the difference when considering WSN nodes calculated position and the real position. For precise localization of nodes, the localization error should be minimized as much as possible. Where (*p*_esti_*i*__, *q*_esti_*i*__) is the unknown node's estimated coordinates, (*p*_*a*_*i*__, *q*_*a*_*i*__) represents the actual positions of an unknown node, and unk represents the number of unknown nodes that have been localized.

(3) $$\begin{array}{c} {\text{LE}}_{i}=\frac{1}{\text{unk}R_{n}}\sum_{i=1}^{\text{unk}}\sqrt{{\left(p_{{\text{esti}}_{i}}-p_{a_{i}}\right)}^{2}+{\left(q_{{\text{esti}}_{i}}-q_{a_{i}}\right)}^{2}}. \end{array}$$

### 3.2. Proposed Algorithm Based on NGC and GA for Localization

In this section, by identifying grid nodes as population centers, we treat the problem as a biological evolution process. Our precise objective seeks to find the target location of a node in a grid with the assistance of a cluster center by choosing the objective function in (2). The problem is solved based on a genetic algorithm (GA) which is adopted with formulated fitness function denoted by FX_*i*_, which consists of energy calculation, node connectivity, and distance estimation.

The following steps describe the genetic improved algorithm embedded with our salient clustering approach can be listed as follows.

#### 3.2.1. Initialization

The first population is formed by a random search, and the initial solution is derived by a genetic operation in the fundamental genetic optimization algorithm. Set the generation counter to *t*=1 and the grid node of the *k*^th^ individual in the *t*^th^ generation to *N*_*i*,*t*_^*k*^. We define *N*_*i*,*t*_^*k*^ as a discrete value and the algorithms accuracy criterion as 1 ≤ *i* ≤ *N*, 1 ≤ *t* ≤ *T*, 1 ≤ *k* ≤ *K*, where *T* signifies the maximum number of generations, and *K* denotes the population size. More exclusively, *N*_*i*,*t*_^*k*^ can be represented as a binary digit sequence. The population of the *k*^*th*^ individual in the *t*^*th*^ generation is denoted as *X*_*t*_^*k*^ and defined as follows:

(4) $$\begin{array}{c} X_{t}^{k}={\left(N_{1,t}^{k},N_{1,t}^{k},\ldots ,N_{N,t}^{k}\right)}^{T}. \end{array}$$

And denoting the total individuals in the *t*^th^ generation, we can obtain the initial population *Y*_1_ as

(5) $$\begin{array}{c} Y_{1}={\left(X_{1}^{1},\ldots ,X_{1}^{K}\right)}^{T}. \end{array}$$

#### 3.2.2. Gene Selection

The selection of individuals for crossover and mutation is biased towards good individuals. By applying a fitness proportionated constructed selection cycle, the gene selection fitness function appoints every fitness cost and subsequently has a likelihood of choosing chromosomes. The individual having a higher fitness value is likely to be selected more. The probability Prob(*X*_*t*_^*k*^) corresponding to *X*_*t*_^*k*^ can be defined as

(6) $$\begin{array}{c} \text{Prob}\left(X_{t}^{k}\right)=\frac{F\left(X_{t}^{k}\right)}{\sum_{k=1}^{K}F\left(X_{t}^{k}\right)}. \end{array}$$

Fitness evaluation is to evaluate the survivability of each individual in the current population. Substitute *X*_*t*_^*k*^ into the fitness for the corresponding value for the fitness, that is, *F*(*X*_*t*_^*k*^) can be obtained. Since we aim to minimize the gene selection fitness function, we pick the members with less Prob(*X*_*t*_^*k*^) from the populace *Y*_*t*_, and reproduction takes place, we can obtain the reproduced population *Y*_*t*_^1^. Avg(*X*_*t*_^*k*^) is the average evaluation of all individuals in the population size *K*.

(7) $$\begin{array}{c} \text{Avg}\left(X_{t}^{k}\right)=\frac{\sum_{k=1}^{K}\left(X_{t}^{k}\right)}{K}. \end{array}$$

#### 3.2.3. Grid Construction Process (Grid Formation)

**Figure 1:**
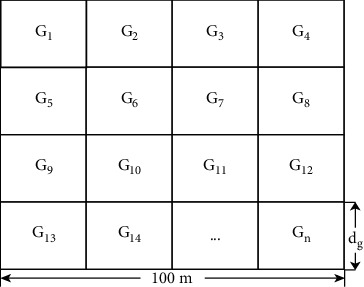
Construction for grid-based clusters.

The methodology of NGC is raised in light of grid division and neighborhood connection whose fundamental idea is derived from the topographies of a sensor field that is partitioned into complete squares. The main steps of NGC are that the sensor nodes are partitioned into grid space, the neighborhood grid density of each grid is acknowledged, and then, adjacent members found in the cluster finally participates based on neighborhood relationship.  Figure 1 shows the grid-based cluster construction. In Figure 1, *d*_*g*_ connotes the side length of each grid *G*_*n*_ where *n*=1 to *n* for a sensing field of 100 m by 100 m. For each grid, the side length *d*_*g*_ is the same. However, *G*_1_ to *G*_*n*_ is the first grid to the last grid. In each grid *G*_1_, there are several nodes and a cluster center.

**Figure 2:**
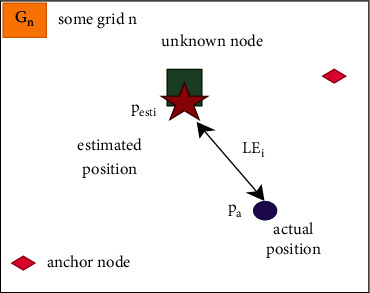
Observed estimated location.

The proposed method considers a sensor network with deployable nodes. Hence, the geographic area is considered to be in a square dimension with several cells referred to as grids. Typically, grid-based clustering protocols consider the nodes inside a similar cell as a group and intermittently select one of the nodes as a cluster center (CC). During the anchor broadcasting stage, CCs are rotated among nodes in a square grid based on categorical models for energy dispersion. Because the sensor network follows arbitrary node arrangement, node dispersal among grid cells is not accomplished by traditional grid-based algorithms. Thus, CC in the heavily populated cell will endure by early energy consumption problem.  Figure 2 displays the observed estimated location.

*(1) Grid Area Estimation*. For a given graph, *G*=(*V*, *E*) with *V* indicates nodes and *E* represents edges (the connection between nodes). Each length of a grid is labeled as *d*_*g*_ with four equal sizes. The entire network is partitioned into *G*_1_ to *G*_*n*_ grids clusters with *d*_*g*_ × *d*_*g*_ equal sizes. Every grid has a set of related nodes. To guarantee that every one of the sensors in the grid network can convey to one another, the length of each side *d*_*g*_ in the grid is calculated using the following equation:

(8) $$\begin{array}{c} d_{g}\leq \frac{R_{n}}{2\sqrt{2}}, \end{array}$$

where *R*_*n*_ denotes the transmission range of a network node. Every grid is given a positive grid identification [*G*_*p*_, *G*_*q*_]. A WSN node *p*_*i*_ can discover the position from its geographic area (*p*_*i*_, *q*_*i*_) which is given as follows:

(9) $$\begin{array}{c} G=\left\{\begin{array}{c} \frac{p_{i}-p_{o}}{d_{g}}=G_{p},\\ \frac{q_{i}-q_{o}}{d_{g}}=G_{q}, \end{array}\right. \end{array}$$

where (*p*_*i*_, *q*_*i*_) denotes the position of a sensor node, (*p*_*o*_, *q*_*o*_) denotes the network's virtual origin during the setup stage, and *d*_*g*_ denotes the grid size. If *G*_*p*_=0 when *p*_*i*_ − *p*_*o*_ < *d*_*g*_ or *G*_*q*_=0 when *q*_*i*_ − *q*_*o*_ < *d*_*g*_ grid display size is zero. To prevent this, we divide *d*_*g*_ by *d*_*g*_/2, until we have *G*_*p*_ ≠ 0 and *G*_*q*_ ≠ 0. The aerial grid is shown by the grid position in the middle. *G*_*p*×*q*_ is an array of grid points, where *p*=1 corresponds to *G*_*p*_ and *q*=1 corresponds to *G*_*q*_.

(10) $$\begin{array}{c} G_{pq}=\left\{p_{o}+\left(p-0.5\right)\times d_{g},q_{o}+\left(q-0.5\right)\times d_{g}\right\}. \end{array}$$

*(2) Grid Cluster Density*. In clustering, it is critical to find the best cluster center node for every grid. To begin, the monitoring space is separated into grids of equal sizes. Each grid has one cluster center node that is responsible for data collection and location routing. Directly or through other CC nodes, the CCs connect with the ground station. The sum of the distances between nodes in the grid is computed by each node in the grid. The same message is sent to all nodes. A grid superior is a node with the smallest sum of distances from other nodes in the grid.

(11) $$\begin{array}{c} \delta_{i}=\frac{dc_{i}}{A_{i}}, \end{array}$$

where *δ*_*i*_ is the neighborhood grid density, *dc*_*i*_ is the node distance *i* from the centroid of the cluster, and *A*_*i*_ is the area denoted as (*πR*_*n*_^2^)_*i*_ with *R*_*n*_ being the communication radius of node *i*. The larger grid size has low *δ*_*i*_. More nodes near the centroid are said to be highly concentrated. In grid clustering, when the area increases with increasing *R*_*n*_, the density of the neighboring nodes is less and vice versa. We assured that in each sequence, only one CC will be chosen per grid. Algorithm 1 is the neighborhood grid clustering method.

#### 3.2.4. Fitness Function Evaluation

In this part, we determine the fitness function for the newly proposed neighborhood grid cluster and location utilizing GA.

*(1) Energy Model*. Energy consumption is a significant issue in the locating of network nodes. Energy is predominantly utilized in message broadcasting, message gathering, and estimating process in localization. The energy model is adopted in this work. The overall energy consumed by a node *i* presented here helps to calculate real energy consumption in models using (12). The energy expended in transitions among states is *E*_*i*_. The amount of energy spent *E*_*Tx*_(*L*, dist_(kn, unk)_) by the node to transmit a packet size of *L* bit length is (13). state_(*k*)_ is the energy condition of the sensor hub during the time period, rest, or transmission.

(12) $$\begin{array}{c} E_{i}=\sum E_{\text{trans}}+{\sum_{{\text{state}}_{\left(k\right)}}\left(P\times t\right)}_{{\text{state}}_{\left(k\right)}}, \end{array}$$

(13) $$\begin{array}{c} E=\left\{\begin{array}{cc} {\text{dist}}_{\left(\text{kn},\text{unk}\right)}<d_{o},\\ \left(E_{\text{Telec}}+\varepsilon_{mp}\cdot {\text{dist}}_{\left(\text{kn},\text{unk}\right)}^{\beta }\right)\cdot L, & {\text{dist}}_{\left(\text{kn},\text{unk}\right)}\geq d_{o}. \end{array}\right. \end{array}$$

However, it is noted that *E*_*Tx*_(*L*, dist_(kn, unk)_)=*E*. The power used up in every state_(*k*)_ is *P*_state_(*k*)__ × *t*_state_(*k*)__ which is the time consumed in the corresponding state. dist_(kn, unk)_ denotes the Euclidean distance among known node kn and unknown node unk. *α* is 2 which represents the free-space channel, and *β* represents the multipath channel which equals 4. *L* is used in transmitting a packet of *L*-bit. Where *E*_Telec_ and *E*_Relec_ is the energy depleted by one sensor node to the other by dispersal and receiving of *L*-bit information, respectively. The threshold distance $d_{o}=\sqrt{\varepsilon_{fs}/\varepsilon_{mp}}$. The energy squandered to receive an information of *L* bits is *E*_*Rx*_(*L*). Also, *ε*_*fs*_ and *ε*_*mp*_ are two constraints of the amplify. *F*_1_ is the energy usage to locate a target node.

(14) $$\begin{array}{c} E_{Rx}\left(L\right)=E_{\text{Relec}}\cdot L,\\ F_{1}=E_{Tx}\left(L,{\text{dist}}_{\left(\text{kn},\text{unk}\right)}\right)+E_{Rx}\left(L\right). \end{array}$$

*(2)* C*onnectivity Model*. A WSN grid is supposed to be covered if each node in the grid cluster falls inside the distance of the sensing radius of a functioning node. However, a network structure is presumed to be connected if any dynamic node can interconnect with some other dynamic nodes inside the grid. The type of sensing model considered is the binary detecting model. Allow *R*_*n*_ to represent the transmitting distance. Two nodes are expected to be connected assuming the distance between them is to such an extent that they can send and get information from one another.

(15) $$\begin{array}{c} R_{n}=\left|\sqrt{{\left(p_{i}-p_{j}\right)}^{2}+{\left(q_{i}-q_{j}\right)}^{2}}\right|, \end{array}$$

where (*p*_*i*_, *q*_*i*_) and (*p*_*j*_, *q*_*j*_) are the coordinates of nodes *i* and *j*, respectively. For a given network scan, the transmitting radius *R*_*n*_ is considered for the entire nodes. Coverage and connectivity assume a significant part in the determination of the size and state of the grid in this technique. If the distance between sensor *p*_*i*_ and *p*_*j*_ is linked, where (*p*_*i*_, *p*_*j*_ ∈ *N*) is within the communication range *R*_*n*_, then *p*_*i*_ and *p*_*j*_ will be termed as being connected. Let *C*_*ij*_ denote the connectivity between *p*_*i*_ and *p*_*j*_. However, *C*_net_ and *C*_*T*_nodes__ are the number of connected nodes and a total number of nodes in the network, respectively. *F*_2_ can be defined as the connectivity strength to find the unknown node.

(16) $$\begin{array}{c} C_{ij}=\left\{\begin{array}{c} 0,\;\left\|p_{i}-p_{j}\right\|\leq R_{n},\\ 1,\;\left\|p_{i}-p_{j}\right\|>R_{n}, \end{array}\right.\\ F_{2}=\frac{C_{\text{net}}}{C_{T_{\text{nodes}}}}. \end{array}$$

*(3) Distance Model*. In a WSN for target localization, the extent of each beacon node is to assess its distance from a given objective node based on the RSS related with the advanced signal transmitted by the actual node. Traditional clustering usually adopts Euclidean distance to measure the distance between two sensor nodes. For each node position in a grid cluster, the uniformity between each position and different locations ought to be determined independently (i.e., the distance from the cluster header). If total distance of point nodes is low, then update to another middle point within every grid cluster. *D*_*in*_ is the average minimum distance between the CCs and the GS (ground station), which is measured as

(17) $$\begin{array}{c} D_{in}=\sum_{i=1}^{G_{n}}\frac{1}{G_{n}}\left|cc_{i}-\text{GS}\right|. \end{array}$$

For the neighborhood representation, a fusion of grid networks is fulfilled when the distances among them and the original centers are under a specific value. The distance defined here is called Euclidean. *D*_out_ is the distance between the cluster nodes to its cluster centers to determine whether the clusters are compact.

(18) $$\begin{array}{c} D_{\text{out}}=\frac{1}{N}\sum_{i=1}^{G_{n}}\sum_{p_{i}\in G_{i}}\left|p_{i}-cc_{i}\right|, \end{array}$$

where *N* is the span of sensor nodes in the system, *G*_*n*_ is the sum of all grid clusters (last grid cluster), and *cc*_*i*_ is the cluster center of grid cluster *G*_*i*_. The effectiveness of clusters is evaluated based on uniformity of node distribution. *F*_3_ is the shortest distance between the two nodes is considered as a fitness function.

(19) $$\begin{array}{c} F_{3}=\sum_{\text{unk}\in N_{\left(\text{kn}\right)}}\left({\text{dist}}_{\left(\text{kn},\text{unk}\right)}\right)=\sqrt{\sum_{d=1}^{D}{\left(p_{\text{kn}}-p_{\text{unk}}\right)}^{2}}. \end{array}$$

Accordingly, the fitness function is developed as follows:

(20) $$\begin{array}{c} {\text{FX}}_{i}=\sum_{i=1}^{3}w_{i}\times F_{i}. \end{array}$$

In the expression above, *w*_*i*_ addresses the weight comparing to the performance value *F*_*i*_ of the fitness function FX_*i*_ where *i*=1,2,3, and thus, the objective function *f*(kn, unk) in (2) is minimized.

#### 3.2.5. Crossover Process

**Figure 3:**
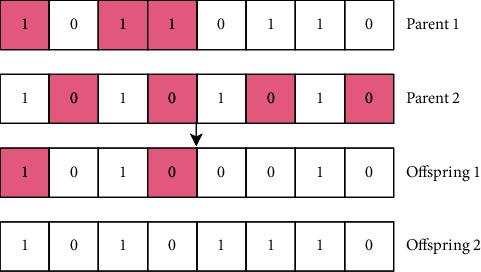
Crossover genetic operator.

In the crossover, reproduction occurs which is mainly between two parents. If no crossover happens, then the offspring will still be the same as the parents. However, the offspring will form parts of the parent chromosome if and only if crossover takes place. Randomly select two parent individuals *X*_*t*_^*k*_1_^, *X*_*t*_^*k*_2_^ from *Y*_*t*_^1^, 1 ≤ *k*_1_, *k*_2_=*K*, pick a number of parent chromosomes from the individuals according to the crossover rate, denoted by *P*_*c*_, then crossover the last few digits of the parent chromosomes to form two children individuals *X*_*t*_*o*__^*k*_1_^, *X*_*t*_*o*__^*k*_2_^ of the next generation through applying scattered crossover-based method. Basically, it makes an irregular paired chromosome and chooses the gene where the chromosome is 1 from the first parent and the gene where the chromosome is 0 from the subsequent parent and later joins the genes to form an offspring.  Figure 3 shows our crossover representation (scattered).

#### 3.2.6. Mutation Process

Mutation may be defined as a small random tweak in the chromosome, to get a new solution. In view of Gaussian transformation process, mutating the two offspring candidates *X*_*t*_*o*__^*k*_1_^, *X*_*t*_*o*__^*k*_2_^ as indicated by specific mutation transformation ratio, signified by *P*_*m*_, and gathering the subsequent candidates *X*_*t*_^*k*^, we can get the last generation. *Y*_*t*_*o*__ represents the next gene after crossover.

(21) $$\begin{array}{c} Y_{t_{o}}=\left(X_{t_{o}}^{1},X_{t_{o}}^{2},\ldots ,X_{t_{o}}^{K}\right). \end{array}$$

**Figure 4:**
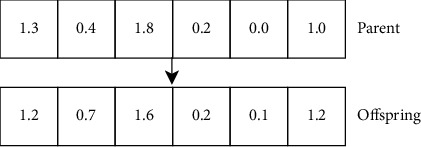
Mutation genetic operator.

Gaussian mutation consists in adding a random value from a Gaussian distribution unit to each element of an individual's gene to create a new offspring. The value is thus added to each element of the gene carriers vector which causes the development of a new offspring.  Figure 4 shows our mutation operator (Gaussian).

#### 3.2.7. Termination Condition

The gene selection fitness values limit is *Y*_*t*_*o*__ if |*F*(*Y*_*t*_*o*__) − *F*(*Y*_*t*_)| ≤ *F*_*th*_, where *F*_*th*_ is the *th* fitness value, the algorithm ends, and *Y*_*t*_*o*__ is compared to a practical candidate solution. When *t*=*T*, the cycle ends and the node localization algorithm stops; otherwise, set *Y*_*t*+1_=*Y*_*t*_*o*__, *t*=*t*+1.

It is worth focusing on the integration of GA which helps put together a strategy depending on the crossover rate, *P*_*c*_, the mutation rate *P*_*m*_, and the fitness function. When the *P*_*c*_ is seen to be higher, a more lesser *P*_*m*_ further develops convergent ratio of the algorithm. Specifically, by picking reasonable *P*_*c*_ and *P*_*m*_, the convergent ratio of the approach is guaranteed. In this paper, we pick *P*_*c*_ and *P*_*m*_ as 0.8 and 0.01, separately, and via simulation, we have confirmed that the gene selection fitness function *F*_*th*_ should be less in a monotonic way with increment in number of iterations. The algorithm accomplishes better convergent ratio with fewer numbers of runs.

#### 3.2.8. Complexity Analysis

The computation complexity of the NGCGAL algorithm proposed is analyzed. The process implored in the algorithm is the neighboring grid cluster which essentially relies on three factors: initial processes, determining the optimal estimated value, and updating solutions. Algorithm 2 requires a complexity computation of *M* × *N* (*M* is the number of iterations, and *N* is the population size) to obtain the initial process of *O*(*M* × *N*). In making decision for nomination in the neighborhood grid clustering, we embrace the intracluster decision-making methodology. The complication of the fitness function is subjected to this problem. At last, the complication of refreshing results is of *O*(*M* × *N*) and *O*((*M*+1)^2^), and *L* is the quantity of boundaries in the problem. Accordingly, the computational analysis of the proposed NGCGAL is of *O*(*N* × *M*(*M*+1))^2^ × *L*.

## 4. Performance Analysis

This segment is partitioned into subheadings. It ought to give a compact and exact portrayal of the simulation results and their understanding, as well as the testing endings that can be drawn.

### 4.1. Simulation Setup

The experiment is deployed on a computer configured as follows: Intel(R) Core (TM) i5-3317U CPU PC with 6144 RAM, which was achieved utilizing MATLAB 2017a simulation platform. The simulator is utilized for comparison between proposed NGCGAL (neighborhood grid clustering in genetic algorithm localization) and prior methods such as DV-hop, CENTA, weighted CENTA, and CGAL.

Distance vector-hop (DV-hop) is one of a progression of circulated localization algorithm called Ad hoc positioning system (APS) in light of distance vector pathfinding. Centroid localization algorithm (CENTA) is a range-free algorithm where the position of a node is found with the help of anchors' coordinates information, and then, the centroid is calculated. The weighted centroid localization algorithm (weighted CENTA) came into existence to provide accurate location estimations as compared to the centroid method where the arithmetic centroid is calculated as the object's location. Clustering in genetic algorithm localization (CGAL) is the normal genetic algorithm extended with clustering methodology which adds to the expansion in positioning exactness.

### 4.2. Experimental Data

Table 1 displays the simulation parameters for the experiment.

### 4.3. Results and Analysis

In this part of the research article, we present the results of implemented algorithm and investigation of these for localization simulation parameters.

#### 4.3.1. Deployment of Sensor Nodes

**Figure 5:**
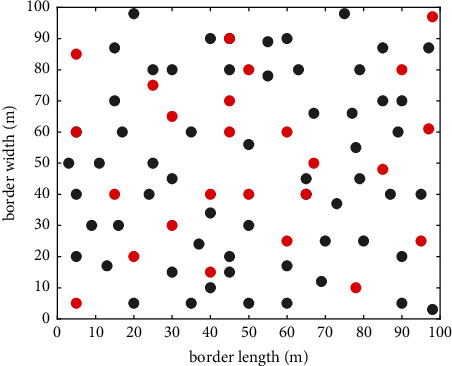
Deployment area of sensor nodes.

In Figure 5, the experiment was performed with randomly deployed sensor nodes. It consists of known and unknown nodes. The localization process is assumed to be carried out for this space. The distance bordering the length area is 100 *m* likewise as the width border. The red dots represent known (anchor) nodes where the locations of their point in the system are certainly known. The black dots are unknown nodes. There are nodes where the location point is undetermined and can be known by calculation using our proposed algorithm.

#### 4.3.2. Number of Formed Clusters Vs Grid Size

**Figure 6:**
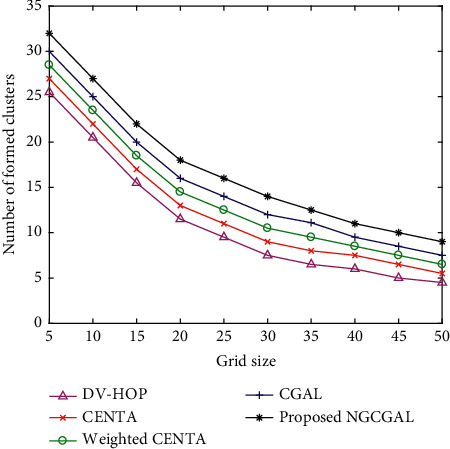
Number of formed clusters against grid size.

In Figure 6, a total 100 WSN nodes are implemented in the simulation field of 100 m by 100 m with approximate divisions of 16 × 16 grids. There are about 8 targets all around in the grid, and their locations are unknown based on theoretical reasons. As the side length of grid increases, the number of clusters that have formed is decreased. When the grid size is 20 m, the number of clusters formed is between 20 and 10 clusters for our proposed approach and the baseline algorithms. The corresponding decline in cluster formation is seen in the proposed NGCGAL compared to CGAL, weighted CENTA, CENTA, and DV-HOP. When the cluster density is low, the area of the grid size is larger with a higher communication radius. If the grid size is excessively large, many clusters may grow within a single grid cell. A single cluster, on the other hand, could be stretched across multiple grid cells if the grid size is small. The larger the grid number *G*_*n*_ is, the more running time NGC takes.

#### 4.3.3. Impact of Increasing Anchor Nodes in Localization Error

**Figure 7:**
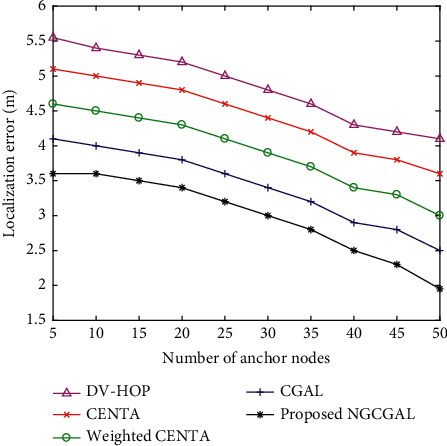
Impact of the increasing number of anchor nodes in localization error.

Figure 7 shows how the localization error changes as the number of anchor nodes rises. There are 100 nodes and 75 m transmission range in a (100 × 100) square-unit region. The number of anchor nodes varies from 5 to 50. Among all localization methods, the proposed NGCGAL achieved the lowest localization error. This is because all prelocalized nodes serve as anchor nodes. When there are about 40 anchor nodes, the error recorded are 2.5, 3.1, 3.7, 4.2, and 4.6, respectively, for the proposed NGCGAL compared to CGAL, weighted CENTA, CENTA, and DV-HOP. The location error for the listed algorithms falls as the ratio of beacon nodes rises in CGAL, weighted CENTA, and CENTA. In DV-hop, because the value of the hop-count decreases, there is an increased density of beacon nodes.

#### 4.3.4. Impact of Increasing Sensing Field in Localization Error

**Figure 8:**
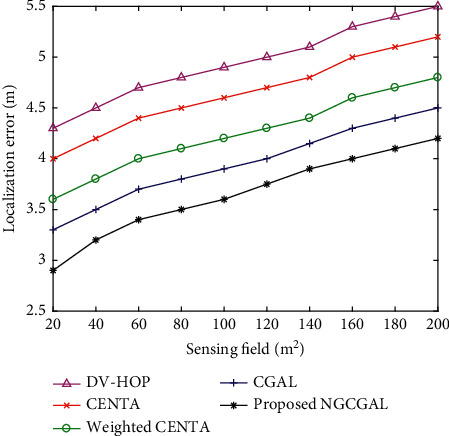
Impact of increasing sensing field in localization error.

Figure 8 shows the variation of localization error with an increase in the deployment area. With about 100 sensor nodes, 40 beacon nodes and 75 m transmission radius have been taken into account. The network deployment area is varied from (100 × 100) m^2^ to (200 × 200) m^2^. As it can be observed from the results, the sensor nodes' localization inaccuracy increases as the deployment area grow for the proposed NGCGAL, CGAL, weighted CENTA, CENTA, and DV-hop. This is because the sensor nodes' transmission range diminishes as the deployment area grows. The connectivity among sensor nodes decreases for weighted CENTA, CENTA, and DV-hop. From the simulated results, it is analyzed that the proposed NGCGAL algorithm accomplishes more accurate localization as compared to other ones.

#### 4.3.5. Impact of Increasing Transmission Range in Localization Error

**Figure 9:**
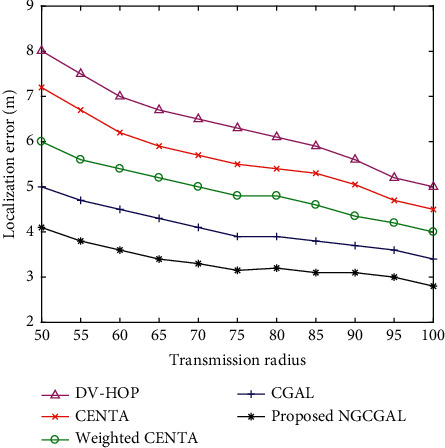
Impact of increasing transmission range in localization error.

Figure 9 depicts the optimal transmission radius ranging from 50 m to 100 m which depends on some number of operators for the minimum location error for the proposed NGCGAL, CGAL, weighted CENTA, CENTA, and DV-hop. The network area is 100 m × 100 m with about 100 sensor nodes. Transmission range is the range of transmitting data between nodes. Nevertheless, note that the localization coverage is unsatisfactory with minimal transmission radius and less number of beacon nodes. In CGAL and weighted CENTA, the strength of a node depletes when transmission of data occurs from one node location to a different node since the transmission range and grid size have a proportional relationship. So, when the grid size decreases, the transmission also decreases. From our graph, when the transmission range extends, the localization error declines which could be seen in CENTA and DV-hop.

#### 4.3.6. Impact of Increasing Number of Nodes in Localization Error

**Figure 10:**
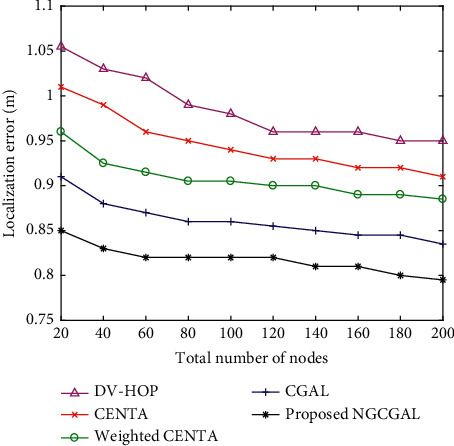
Impact of increasing number of nodes in localization error.

Figure 10 illustrates that as the number of system nodes amasses, the grid density of nodes also increases, and when this happens, the anchor nodes primarily communicate in order to participate in the positioning, and the positioning error of the five algorithms is decreased. The simulation environment considered increasing nodes from 20 to 200 within the predefined area of 100 m^2^. Whenever the level of nodes in the network is reduced, the most shortest way is confounded, and the aggregated distance error is enormous, so the error of DV-hop is more greater than the proposed algorithm. The weighted CENTA utilizes weights to find the minimum base number of hops between nodes as the most limited way, and the method is to track down the way with the shortest distance. In CENTA, when the quantity of nodes in the network expands, the most direct way is nearer to the real distance between nodes than the shortest hop route. Notwithstanding, the assessment of distance has an incredible connection with the neighborhood grid density of nodes.

#### 4.3.7. Impact of Increasing Number of Iterations in Energy Consumption

**Figure 11:**
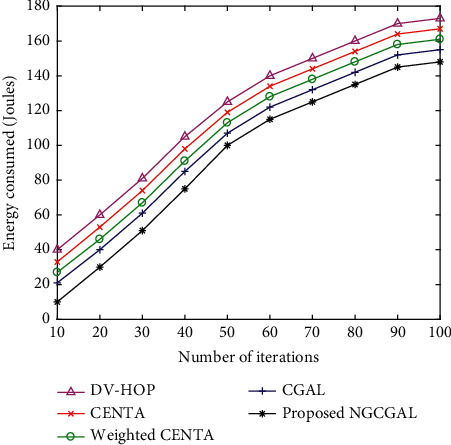
Impact of increasing number of iterations in energy consumption.

Figure 11 demonstrates the energy consumed plotted against the number of iterations. With 100 iterations, the proposed NGCGAL approach studies the distance path of neighboring nodes in the location nearest to the ground station (GS). These sensor nodes are important for the network to interconnect straightforwardly with the GS, which diminishes the excessive computational time for sending information by means of CC nodes. Hence, the energy distribution of the network is more balanced. The number of iterations maximises with the energy consumed in joules also adding up. There are 100 network nodes. CGAL, weighted CENTA, and CENTA upgrade the node grouping strategy, and choosing process for grid cluster reproduction, and between cluster information transmissions, the energy utilization of the nodes can be adjusted successfully, and the system network lifetime is broadened. Contrasted with the DV-hop approach, the network activity period is diminished and is less productive than the proposed NGCGAL algorithm because of the abandoned cluster arrangement. At last, the switch off and rest mode shield the sensor node from the wasteful transmission and cluster center from an inactive listening stage.

#### 4.3.8. Impact of Increasing Number of Iterations in Number of Alive Nodes

**Figure 12:**
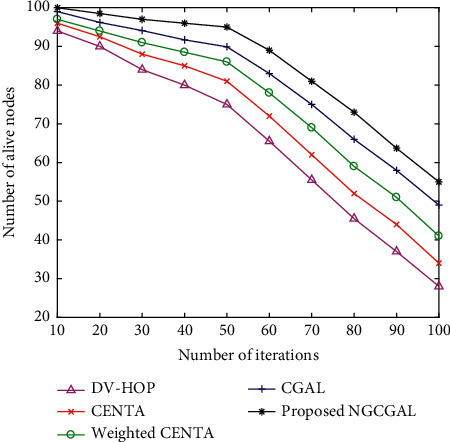
Impact of increasing number of iterations in number of alive nodes.

Figure 12 presents the network lifetime of the system. To evaluate network lifetime, we simulate a network with 1 J for each node as the initial energy and stop the simulation when no nodes are able to transmit directly to the system within 100 m^2^. The system network lifetime is a significant thought of WSN. As the number of alive nodes decreases gradually for the proposed NGCGAL, CGAL, weighted CENTA, CENTA, and DV-hop, the number of iterations increases. In the proposed NGCGAL, the cluster centers can perform data aggregation for communicating to all the specified final destinations. Even with the average drop in the speed of convergent of the proposed NGCGAL assessed with other baseline approaches, it, however, has the improvement of dealing with the entire domain of outcomes for a total of 100 sensor nodes.

#### 4.3.9. Impact of Increasing Connected Nodes in Coverage

**Figure 13:**
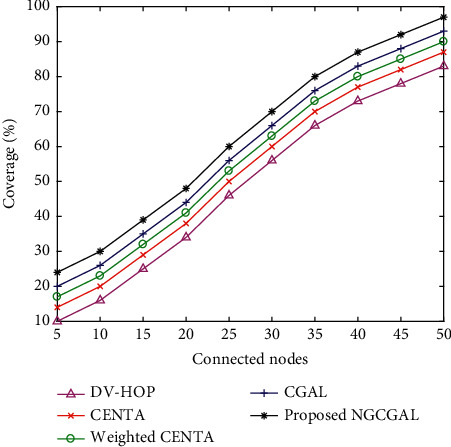
Impact of increasing connected nodes in coverage.

Figure 13 shows the impact of connectivity on connected nodes. In this research article, the concept of connectivity of positioned nodes is used to evaluate the network grid. To analyze coverage and connectivity, connected nodes are varied as network connectivity from 5 to 50 with 100 nodes. When the known nodes are more and the transmission range is increased, it is expected that there are more connectivity identified among the nodes. For all the algorithms, the number of nodes including known and unknown was randomly distributed in a fixed field 100 m^2^. The target area is assumed to be well defined within the grid. In the proposed NGCGAL, CGAL, weighted CENTA, and CENTA are all classified as clustering algorithms; therefore, when all node positions are fixed, the location point with communication radius is improved by an effective connectivity model. As the number of connected nodes increases for the proposed NGCGAL, CGAL, weighted CENTA, CENTA, and DV-hop, the coverage area also increases.

## 5. Conclusion and Future Work

The proposed clustering algorithm based on neighborhood grid clustering structure solves the localization problem. However, when locating a target node, new nodes are generated from GA after selection, and crossover and mutation calculate the distance in order to find the estimated position. The fitness function in this technique is constructed using an energy model, distance measurement, and connectivity estimation. A broad experimental analysis of the algorithm under various situations has been introduced. Compared with CGAL, weighted CENTA, CENTA, and DV-HOP, the outcome for the proposed NGCGAL shows that the localization approach implemented outperforms the supplementary four baseline methodologies for different constraints.

However, several research drawbacks exist, such as the nodes constraint, distance error, and mobility. Therefore, in the future, we will try to focus on the integration of IoT and WSN for real-time localization systems using smart connected sensors for innovative IoT applications. The location aspect of home aid robots and driving assistance can be investigated with advanced swarm/artificial intelligent algorithms.

## Figures and Tables

**Algorithm 1:**
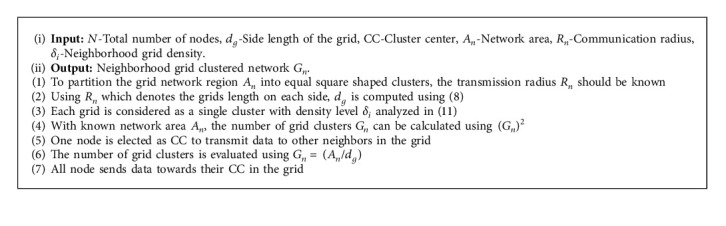
Algorithm for the neighborhood grid clustering approach.

**Algorithm 2:**
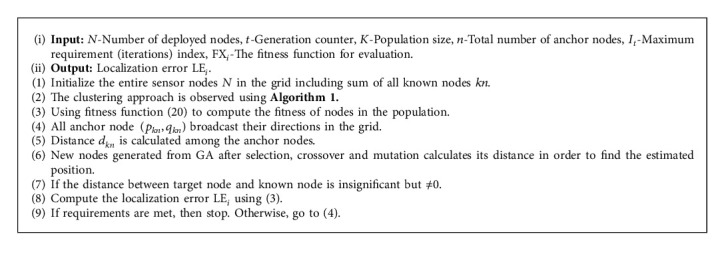
Pseudocode for localization algorithm based on NGC and GA.

**Table 1:** Simulation parameters.

Parameters	Values
WSN area *A*_*n*_	100 × 100 sq.metres
Total nodes *N*	100
Anchor nodes kn	40
Number of unknown nodes unk	*N* − kn
Transmission range *R*_*n*_	75 m
Side length of grid *d*_*g*_	≤26.52
Network style	Grid-based
Maximum iterations *I*_*t*_	100
Population size *K*	50
Crossover rate in GA *P*_*c*_	0.8
Mutation rate *P*_*m*_	0.01
Number of clusters	15–17
Chromosome length	5
Evolutional algorithm	GA
Number of generations *t*	150
Number of ground stations GS	1
Coverage area	95 metre^2^
Connection type	UDP
Initial energy	1 J/battery
Distance *d*_*o*_	87 m
Data packet length	4000 bits
Transmission speed	400 bit/s
*E*_Telec_/*E*_Relec_	50 pJ/bit
*ε*_*fs*_	10 (pJ/bit)/m^2^
*ε*_*mp*_	0.0013(pJ/bit)/m^4^
Propagation model	Free-space/Multipath fading
Network simulator	MATLAB
*w*_1_, *w*_2_, *w*_3_	*w*_1_+*w*_2_+*w*_3_=1

## Data Availability

The (deplodata pdf) data used to support the findings of this study are included within the supplementary information file.

## Supplementary Materials

Supplementary Materials In the daplodata.xlsx, *N* is the total number of nodes, and kn is the number of known nodes. *A*1 is the data number, which is used in this paper from 1 to 60. *B*1 is the distribution of the *X*-axis of the data, and in this paper, the maximum range of the *X*-axis is 100 m. *C*1 is an unknown node whose number is equal to *N* − kn. *D*1 is a known node whose number is kn. *E*1 is nodes beyond the range of deployment space (rendered null).
